# Biotic and Abiotic Factors Controlling Respiration Rates of Above- and Belowground Woody Debris of *Fagus crenata* and *Quercus crispula* in Japan

**DOI:** 10.1371/journal.pone.0145113

**Published:** 2015-12-14

**Authors:** Mayuko Jomura, Yuhei Akashi, Hiromu Itoh, Risa Yuki, Yoshimi Sakai, Yutaka Maruyama

**Affiliations:** 1 Department of Forest Science and Resources, College of Bioresource Sciences, Nihon University, Fujisawa, Kanagawa, Japan; 2 Kyusyu Research Center, Forestry and Forest Products Research Institute, Kumamoto, Kumamoto, Japan; DOE Pacific Northwest National Laboratory, UNITED STATES

## Abstract

As a large, long-term pool and source of carbon and nutrients, woody litter is an important component of forest ecosystems. The objective of this study was to estimate the effect of the factors that regulate the rate of decomposition of coarse and fine woody debris (CFWD) of dominant tree species in a cool-temperate forest in Japan. Respiration rates of dead stems, branches, and coarse and fine roots of *Fagus crenata* and *Quercus crispula* felled 4 years prior obtained *in situ* ranged from 20.9 to 500.1 mg CO_2_ [kg dry wood]^–1^ h^–1^ in a one-time measurement in summer. Respiration rate had a significant negative relationship with diameter; in particular, that of a sample of *Q*. *crispula* with a diameter of >15 cm and substantial heartwood was low. It also had a significant positive relationship with moisture content. The explanatory variables diameter, [N], wood density, and moisture content were interrelated. The most parsimonious path model showed 14 significant correlations among 8 factors and respiration. Diameter and [C] had large negative direct effects on CFWD respiration rate, and moisture content and species had medium positive direct effects. [N] and temperature did not have direct or indirect effects, and position and wood density had indirect effects. The model revealed some interrelationships between controlling factors. We discussed the influence of the direct effects of explanatory variables and the influence especially of species and position. We speculate that the small *R* ^2^ value of the most parsimonious model was probably due to the omission of microbial biomass and activity. These direct and indirect effects and interrelationships between explanatory variables could be used to develop a process-based CFWD decomposition model.

## Introduction

As a large, long-term pool and source of carbon, woody litter is an important component of forest ecosystems’ carbon budgets. The amount of carbon stored in woody litter is estimated to account for 17% of total forest biomass worldwide [1] and 5%, 18%, and 10% in boreal, temperate, and tropical forests, respectively [2]. The amount of woody litter can greatly increase after disturbance by insect attack [3], forest fire [4], and hurricanes [5], in addition to the natural tree mortality caused by competition or succession. Climate change may increase the frequency of disturbances [6], increasing the amount of woody litter in forests. During the decay of woody litter, most of the carbon is respired to the atmosphere as a consequence of microbial activity. Because decomposition of woody litter can take years to decades, woody litter acts as a long-term source of carbon release to the atmosphere. Although respiration from snags and logs is generally not a large component of total ecosystem respiration [7, 8, 9], a large amount of woody litter would reduce the strength of the carbon sink in many forests [10]. Modeling of the respiration rate of woody litter, therefore, will allow for accurate estimation of forest carbon budgets and the global carbon cycle.

Woody litter includes stems, branches, and roots of a range of sizes. Coarse and fine woody debris (CFWD) decomposes aboveground as snags and attached branches, on the ground as logs and fallen branches, and in the soil as stumps and roots [11]. Inputs of CFWD are heterogeneous and irregular, and decomposition can take years to decades, even centuries [12]. Therefore, multiple biotic and abiotic factors affect the rate of CFWD decomposition. Factors include physical properties of wood such as density and diameter [3, 4, 9, 13, 14, 15]; chemical factors such as concentrations of carbon ([C]) and nitrogen ([N]), C:N ratio, and lignin:N ratio [10, 16]; environmental factors such as moisture content and temperature [7, 9, 13, 17, 18, 19, 20, 21, 22, 23, 24, 25, 26, 27, 28]; time since death and decay class [8]; species, parts, and position of woody debris [8, 29]; and fungal species composition [30, 31]. Interrelationships between variables have also been observed. For example, wood moisture content is strongly related to wood density [13, 32], wood diameter and [N] [12], and [C] and wood specific gravity [33]. Therefore, to model the decomposition of woody litter, we should consider various direct and indirect effects of the controlling factors and the effect of interrelationships between variables.

The physical structure of woody organs, which is related to or regulates other chemical or environmental factors [12], complicates the investigation of the effects of controlling factors on the woody litter decomposition rate. Macro- and micro-scale structures of wood are associated with the decomposition rate of woody litter [11, 12, 34]. Because the structure of xylem, the main component of wood, and other components varies among species and between angiosperms and gymnosperms [34], wood density varies according to the size and arrangement (e.g., diffuse, ring, or radial-porous wood) of tracheids or vessels [35, 36]. Root wood has different characteristics from stem wood, presumably related to differences in their structure and function, such as larger and more numerous vessels with thinner walls [12], but data on root wood are limited compared with data on stem wood [37]. The physical properties are related to chemical and environmental factors. The C:N ratio and the lignin:N ratio increase with increasing diameter because of the high [N] in bark and phloem [12]. Because heartwood has compounds resistant to decomposition, such as tannins, and sapwood has living parenchyma with easily decomposable carbon compounds such as sugars [11, 34], the increase in the proportion of heartwood with increasing diameter [38] would be expected to influence the relationship between the size and decomposition rate of woody litter. Wood density is strongly related to moisture content [13], and water-holding capacity would change with decomposition by microbes or borers [12]. Some controlling factors are related to or are regulated by the physical structure of woody debris, but their relative strengths and interrelationships have rarely been determined in relation to the decomposition rate of woody debris. We devised this study to determine the direct and indirect effects of these factors on the respiration rate of woody litter.

The influence of various biotic and abiotic factors on microbial respiration *in situ* is difficult to predict because of the wide variation in environmental factors on daily and seasonal scales [9]. Laboratory experiments allow factors to be controlled, but recent research has shown that microbial biomass and enzyme dynamics differ significantly between laboratory and field conditions [39]. Under field conditions, some factors can be controlled: for example, samples felled at the same time can be used to set time since death or decay class; using stems, branches, and roots cut at the same time can clarify the effect of organ; and a one-time measurement in a specific season can eliminate seasonal temperature effects on respiration rate.

In this study, we measured the respiration rate of above- and belowground CFWD while controlling for the effects of time since death and temperature by using trees felled at the same time and a one-time measurement in summer, and evaluated the effects of other factors on the rate of CFWD respiration. We investigated how physical, chemical, and environmental factors affect CFWD respiration rate directly and indirectly; how tree species and position influence CFWD respiration rate through physical, chemical, and environmental factors; and what could explain the rest of the variance in CFWD respiration rate.

## Materials and Methods

### Site description

The study was conducted in Nihon University’s Minakami Forest (36.8°N, 139.0°E; 850 m a.s.l.). The vegetation is cool-temperate deciduous forest dominated by *Fagus crenata*, *Quercus crispula*, *Acer mono*, *Magnolia hypoleuca*, and *Betula grossa*. The soil is a Brown Forest soil. The mean annual air temperature was 10.2°C and the mean annual precipitation was 1742.4 mm from 1979 to 2008 at the Minakami weather station, about 5 km from the site (National Climate Center). Snow is present from December to April at a typical depth of 1 m. At the time of the study the tree density (diameter at breast height: DBH > 3 cm) was 1020 ha^–1^ and the mean DBH was 18.4 cm. Tree ring counts indicated that most of the trees are >60 years old.

### Sample preparation and measurements

In November 2008, five trees each of *F*. *crenata* and *Q*. *crispula*, which are dominant species in the cool temperate region in Japan, were cut down and left on the ground. In August 2012, aboveground parts (*n* = 40, 1.6 cm < diameter [*D*] < 18.0 cm) of each dead tree were collected and cut into lengths of 80 cm (*D* ≥ 15 cm) or 30 cm (*D* < 15 cm). Roots were excavated in June (*F*. *crenata*) and July (*Q*. *crispula*) of 2012 and reburied to permit measurement on the same date later in the summer. In August 2012, a day before the measurements, root samples of each tree were dug out and cut into lengths of 30 cm (*D* ≥ 1 mm) or 10 cm (*D* < 1 mm) (*n* = 50, *D* < 63 mm). Samples with a diameter of <1 mm were wrapped in 1-mm nylon mesh to avoid loss by fragmentation. The cut surfaces of all samples were sealed with silicone sealant to prevent CO_2_ emission from them, and the samples were again reburied.

The following day, which was a fine day with a range of ambient temperature between 26 to 34°C, respiration rates of dead aboveground and dug-up belowground samples were measured *in situ*. First, their fresh weights were measured on spring or digital scales, and their diameters and lengths were recorded by digital Vernier caliper and scale. Then they were placed in airtight acrylic chambers (60 830, 5000, or 304 cm^3^, depending on the size of the sample), and the CO_2_ concentration in the headspace was measured with an infrared gas analyzer (GMP343, Vaisala Inc.) from 1 to 5 min after closure of the chamber. The temperature inside the chamber and the ambient temperature were measured using temperature probes. Data were recorded on a data logger (GL200A Graphtec Corp.) every 1 s.

After the respiration measurements, 3-cm-thick cross-sections of large samples (*D* ≥ 15 cm) were obtained by chainsaw. The cross-sections and the remaining samples were dried in an oven at 65°C to constant weight and weighed.

From the data and the temperature in the chamber we calculated wood moisture content (g H_2_O g^–1^ dry wood), volume (cm^3^), density (g cm^–3^), temperature in the chamber, and dry-weight-based respiration rate (mg CO_2_ [kg dry wood]^–1^ h^–1^). Samples were assumed to be a cylinder to calculate volume. If heartwood was observed on cross-sections, heartwood diameter was recorded and sapwood ratio was calculated for the area basis. Sapwood ratio assumed to be 100% for the samples in which heartwood was not observed. The entirety of each sample was then ground in a mill, and two replicate measurements of [C] and [N] were determined with a CN analyzer (Sumigraph NC analyzer NC-22F, NC-220F, Sumika Chemical Analysis Service, Ltd.). Mean properties of samples at each position in each species were shown in Table 1. (Raw data of respiration rate and physicochemical and environmental factors are presented in S1 Appendix).

**Table 1:** Diameter, wood density, moisture content, carbon and nitrogen concentrations, and respiration rate of CFWD at each position in each species.

Species	Position	*n*	Diameter	Wood density	Water content	C conc.	N conc.	Respiration
			cm	g cm^–3^	g g^–1^	%	%	mg CO_2_ kg^–1^ h^–1^
*Fagus crenata*	aboveground	20	7.9(1.6–17.0)^a^	0.30(0.16–0.47)^a^	1.14(0.42–1.98)^a^	46.1(45.2–46.9)^a^	0.29(0.12–0.73)^a^	109.2(22.4–193.5)^a^
	belowground	25	2.1(0.2–5.8)^b^	0.51(0.27–0.90)^b^	1.62(0.93–2.79)^b^	48.2(46.6–47.0)^b^	0.38(0.17–0.56)^a^	96.0(29.1–197.3)^a^
*Quercus crispula*	aboveground	20	8.3(1.7–18.0)^a^	0.39(0.21–0.66)^b^	0.98(0.38–2.00)^a^	46.1(44.8–47.2)^a^	0.29(0.08–0.59)^a^	115.9(20.9–500.1)^a^
	belowground	25	1.8(0.1–6.3)^b^	0.58(0.38–0.93)^c^	0.97(0.61–1.51)^a^	48.7(47.7–49.8)^b^	0.43(0.27–0.59)^b^	110.6(31.8–282.7)^a^

Values in parentheses are minimum and maximum values.

Mean values with same letters in a column are not significantly different (*P* < 0.01).

### Statistical analysis

All statistical analyses were performed in the R statistical package (v. 3.1.2, R Development Core Team). The distributions of the diameter, temperature, and C:N ratio data were skewed, so the data were log-transformed for analysis.

Differences in CFWD properties and respiration rates between species and positions were tested by ANOVA followed by Tukey’s HSD *post hoc* test. We computed Pearson’s correlation coefficients for the relationships among all properties and respiration rate.

**Fig 1:**
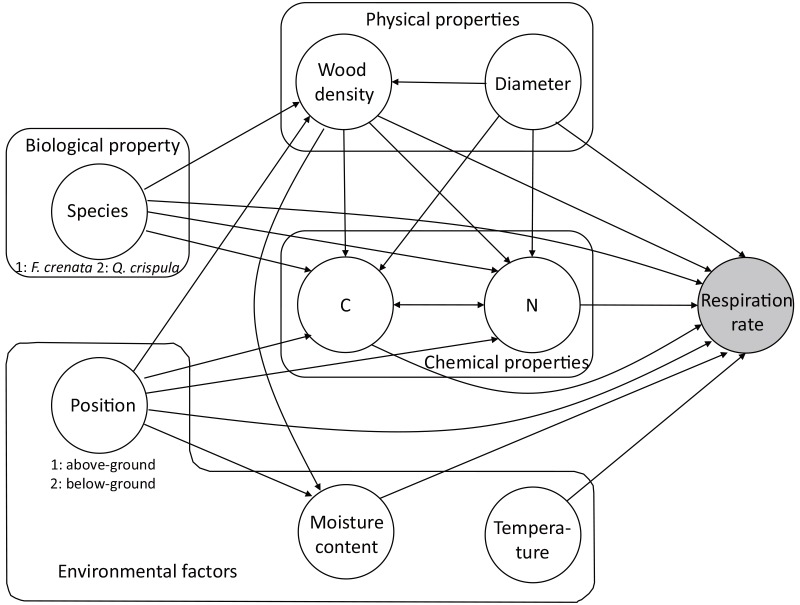
A priori path model representing anticipated relationships among variables.

We used path analysis [40], which allows the evaluation of complicated interactions, to examine the control of CFWD respiration rate by the factors. We considered an *a priori* model of pathways that we believed represented processes operating in the decomposition of CFWD (Fig 1). On the basis of the results given by the *sem* package in R [41], we eliminated non-significant variables and pathways until there were no non-significant pathways. Then we added significant interrelations indicated by the modIndecis function in the *sem* package to obtain the most parsimonious model. Values of standardized path coefficients indicate effect sizes: typically, <0.1 = small, ~0.30 = medium, and >0.5 = large [40]. The most parsimonious path model used tree species (1, *F*. *crenata*; 2, *Q*. *crispula*) as a biological factor; position (1, aboveground; 2, belowground), moisture content, and temperature as environmental factors; wood density and diameter as physical factors; and [C] and [N] as chemical factors.

## Results

**Fig 2:**
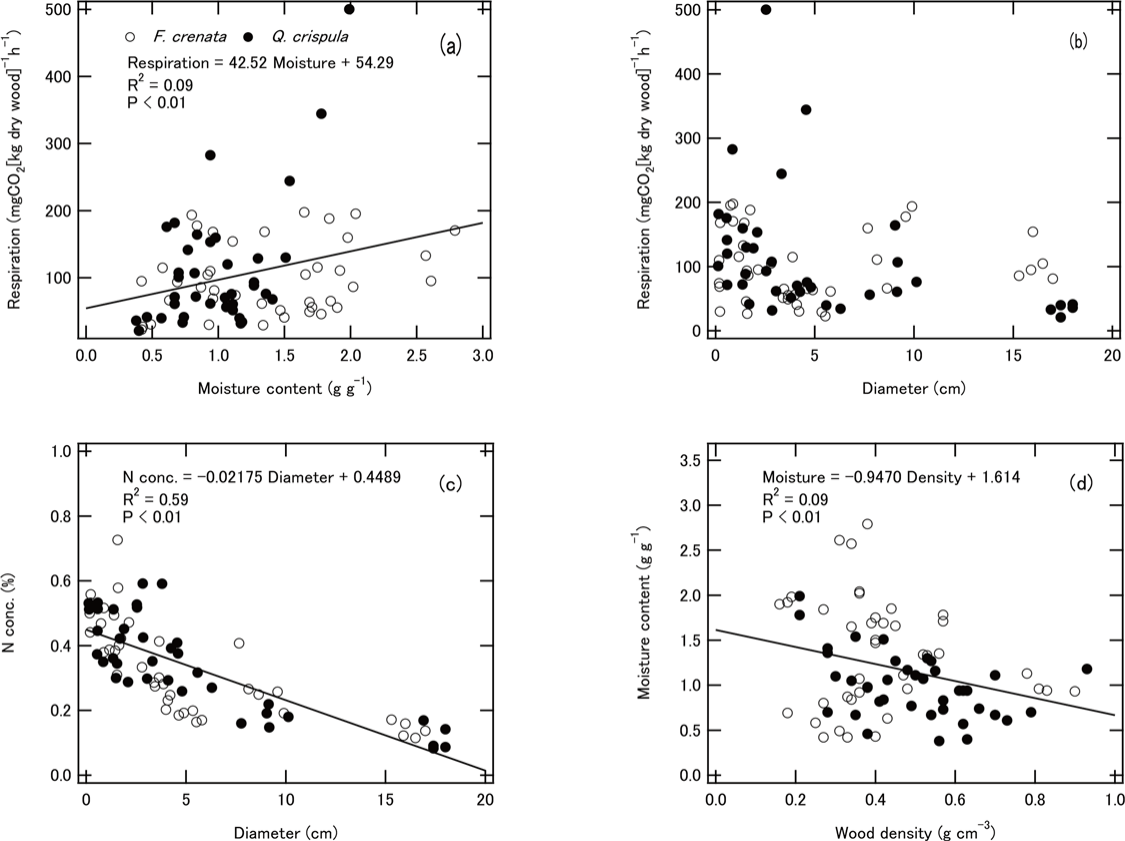
Relationships between (a) moisture content and respiration rate, (b) diameter and respiration rate, (c) diameter and [N], and (d) wood density and moisture content. ○ *F*. *crenata*; ● *Q*. *crispula*.

The physicochemical properties and respiration rate at each position in each species are shown in Table 1. Diameter differed significantly between positions: aboveground samples were larger in both species. Wood density differed significantly between positions and between species: *F*. *crenata* had lower density than *Q*. *crispula*, and aboveground samples had lower density than belowground samples. The moisture content of belowground samples of *F*. *crenata* was significantly higher than that of aboveground samples, but those of *Q*. *crispula* did not differ. [C] differed significantly between positions in both species, but [N] differed significantly only in *Q*. *crispula*. Individual CFWD respiration rates ranged from 20.9 to 500.1 mg CO_2_ [kg dry wood]^–1^ h^–1^ within a temperature range of 24.1 to 36.2°C but did not differ significantly between species or between positions (Table 1). Respiration rate tended to increase with moisture content (Fig 2A), more clearly in *F*. *crenata* that in *Q*. *crispula*; it was not significantly correlated with diameter (Fig 2B). A sample of *Q*. *crispula* with a diameter of >15 cm had heartwood (83% of the total cross-sectional area) that had a significantly lower respiration rate than one of *F*. *crenata* of the same size with no heartwood (*t*-test, *P* < 0.01). [N] tended to decrease with increasing diameter (Fig 2C), and wood density was significantly negatively correlated with moisture content (Fig 2D).

**Fig 3:**
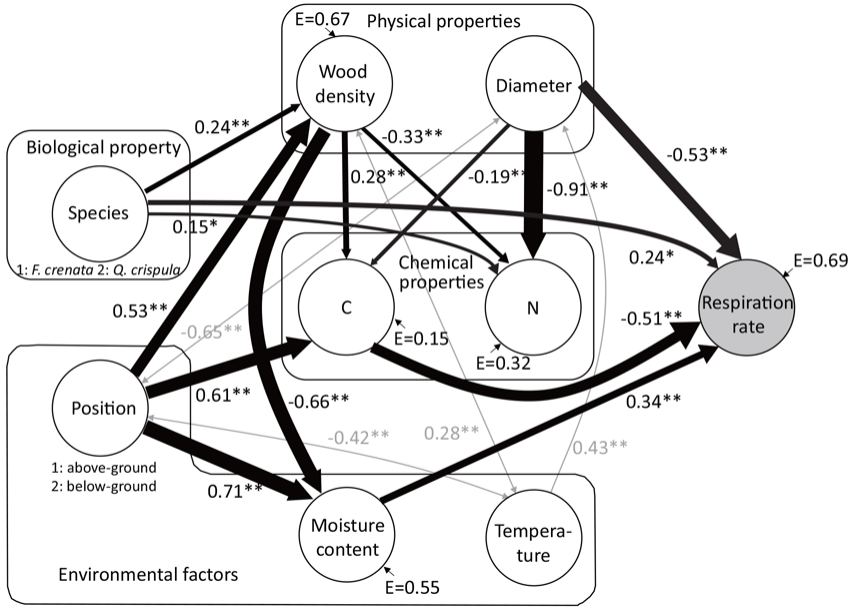
Path diagram explaining biological, physical, chemical, and environmental factors that influence CFWD respiration rate. Standardized path coefficients are given on the path arrows: *P < 0.05; **P < 0.01. Solid paths are positive; broken paths are negative. Gray paths indicate artificial correlations caused by our experimental design; these paths were excluded from consideration. E = error variance.

Some pairs of factors showed correlations (Table 2), so we analyzed the effects of these variables on the variation in respiration rate by using path analysis. The final best-fit path model incorporated 14 significant paths between 8 factors, all of which passed all statistical tests of adequacy (*P* = 0.25, goodness of fit = 0.93, comparative fit index = 0.99, root mean square error of approximation = 0.05; Fig 3). The model explained 31% (100% minus standardized error variance of 69%) of the variance in respiration rate. Diameter and [C] had large negative direct effects on respiration rate, and moisture content and species had medium positive direct effects (Fig 3). [N] and temperature did not have direct or indirect effects, and position and wood density had indirect effects. The model also revealed interrelationships between factors. The effects of direct explanatory variables, indirect variables (species and position), and unknown factors on respiration rate are discussed in the following sections.

**Table 2:** Pearson’s correlation coefficients for the relationships between pairs of CFWD variables.

	Species	Position	Diameter	Moisture	Temperature	Density	[C]	[N]	Respiration
Species	1.00								
Position	–0.06	1.00							
Diameter	0.04	–0.65**	1.00						
Moisture	–0.34**	0.37**	–0.13	1.00					
Temperature	0.12	–0.43**	0.40**	–0.36**	1.00				
Density	0.24*	0.51**	–0.41**	–0.30**	0.05	1.00			
[C]	0.02	0.88**	–0.70**	0.07	–0.26*	0.67**	1.00		
[N]	0.04	0.42**	–0.76**	0.21	–0.36**	0.08	0.40**	1.00	
Respiration	0.10	–0.02	–0.21	0.30**	–0.11	–0.26*	–0.11	0.29**	1.00

**P < 0.01

*P < 0.05.

## Discussion

### Factors directly controlling respiration rate

Diameter had a large negative direct effect on respiration rate (Fig 3). Previous studies showed conflicting effects of diameter on the decomposition rate of woody debris [16, 42, 43]. The most appropriate explanation for the negative effect in our results is that the decrease in surface area to volume ratio with increasing diameter restricted fungal access, thus decreasing the decomposition rate [3]. This would be especially true in the early stage of decomposition, before microbes had colonized the whole wood. The relationship between diameter and surface area to volume ratio, which affects on the rate of fungal colonization, follows a negative power function. The decrease in the ratio with increasing diameter is faster at smaller diameters than at larger diameters, indicating that the clear negative effect of diameter on respiration rate was due to our inclusion of samples with a diameter small as 0.1 cm (Table 1). Larger stems can include heartwood, which usually decomposes more slowly than sapwood [5]. *Q*. *crispula* with *D* > 15 cm in which heartwood was observed had a significantly smaller respiration rate than that of *F*. *crenata* of the same size (*t*-test, *P* < 0.01). We added sapwood ratio as an explanatory variable in the path analysis, as discussed in the next section. The negative effect of diameter on respiration rate would relate to fungal access and heartwood content. However, larger woody debris can sometimes decompose faster than smaller debris because of the increase in the internal surface area caused by borers, and because the timing of falling to the ground depends on size [42, 43].

[C] had a large negative direct effect on respiration rate (Fig 3). Structural and secondary compounds have a high [C]—63% to 66% in lignin and >70% in tannins [44]—and they decompose more slowly than cellulose ([C] = 44%, [44], [45]). Negative direct effect of [C] on respiration rate would be due to the difference in the composition of chemical compounds. Path analysis using C:N ratio as a controlling factor showed that it did not have either direct or indirect effects on the rate of CFWD respiration (data not shown), so it was not suitable as a chemical factor to explain the variance in CFWD respiration rate. [C] and [N] were controlled by different driving factors in wood: wood is high in chemical components with high [C] [44], but the inner bark in live stems and roots has high [N] [46, 47, 48]. Because the C:N ratio offset these properties, [C] and [N] should be used separately as an explanatory variable for CFWD decomposition. Surprisingly, [N] did not have a direct effect on respiration rate (Fig 3), though [N] is an important factor in decomposition [10].

Moisture content had a medium positive effect on respiration rate (Fig 3). The moisture content of wood affects the growth of wood-decaying fungi in two ways, as water supply is restricted at low moisture content, and the water-filled void space limits gaseous exchange [12]. In this study, we did not observe limitations to respiration rate at high moisture content, even in belowground samples. The effect of wood moisture content is discussed in the next section.

The direct medium effect of species on respiration rate suggests hidden factors unrelated to those we measured, such as fungal species composition specific to wood species. The positive coefficient suggests that one species (*Q*. *crispula*) had more active decomposers than the other species (*F*. *crenata*). Because fungal species succession *in situ* is reported only for *F*. *crenata* [49, 50], the specificity of decomposers for these species could not be compared. However, fungal communities generally differed among tree species [51] and dissimilarity of species composition between logs was higher in earlier decay classes [50]. As our samples were in the earlier stages of decomposition, fungal species specific to *Q*. *crispula* probably resulted in the higher respiration rate of *Q*. *crispula* (Table 1).

### CFWD Species and components

Species had three routes of effect on respiration rate with opposing actions: a medium positive direct effect and small negative indirect effects intermediated by wood density and [C] and by wood density and moisture content (Fig 3). The direct effect of species is discussed in the previous section. Species had a medium positive effect on wood density, indicating that *Q*. *crispula* had higher wood density than *F*. *crenata*. The higher wood density produced a lower moisture content, which decreased the respiration rate. It is commonly observed that wood moisture content increases with decreasing wood density of CWD [4, 13, 14], as our results show. Decomposers increases the void space while decreasing the density, and the available void space determines the water-holding capacity of dead wood [12]. As the moisture content of CFWD was directly related to respiration rate, wood density would be a good factor for prediction of the moisture condition of woody litter.

The high wood density also related to the high [C], which was associated with lower respiration rate (Fig 3). In general, *Q*. *crispula* has a higher wood density than *F*. *crenata* [52], probably because *Q*. *crispula* makes more heartwood [52] with more tannin (C% > 70%, [44] than *F*. *crenata* [53]. The decomposition of samples increased in the order of *Q*. *crispula* heartwood < *F*. *crenata* heartwood < *Q*. *crispula* sapwood [54], suggesting that the decomposability of these species depends on the sapwood ratio which related to wood density and [C]. Our aboveground samples of *Q*. *crispula* (*D* > 15 cm) had heartwood and showed a lower respiration rate than that of *F*. *crenata* (*t*-test, *P* < 0.01). However, the addition of sapwood ratio as an explanatory variable did not improve the model (data not shown). Although sapwood ratio was not a prominent factor controlling the CFWD respiration rate in the model, chemical properties represented by wood density would be important factors in the effect of species on respiration rate.

Position did not have a direct effect on respiration rate (Fig 3), suggesting that there were no position-specific effects such as a difference in fungal community compositions between positions. Hood et al. [55] found more fungal species in aboveground dead wood than in belowground dead wood. Differences in fungal spore dispersion patterns and in gaseous and moisture conditions between above and below the soil surface would be expected, but a difference in respiration rate related to position was not prominent in our results.

Position had large direct effects on [C] and on wood density (Fig 3), suggesting that belowground samples had different structural and chemical properties, represented by the higher [C] and wood density than those of aboveground samples (Fig 3, Table 1). As root wood density and [C] measurements are rare, differences in wood density and [C] between positions are unclear. For example, Kuyaha et al. [56] showed that root wood density was smaller than stem and branch densities, whereas Namm and Berrill [37] showed that root wood density was not significantly different from that of stumps, and found a greater [C] in the root system than in stumps. Above- and belowground decomposition rates should be compared in terms of structure and chemical properties.

Position also had a large positive effect on moisture content (Fig 3), suggesting that moisture was greater in the belowground parts than aboveground ones. Dead wood moisture content is governed by wetting and drying, but drying below ground is limited. To determine whether the effect of position on moisture content is temporary or normal, future studies should monitor the effect of position on moisture content. The model indicated that the balance between the opposing effects of position intermediated by moisture content, [C], and wood density would determine the decomposition rate of above- and belowground parts, although previous studies did not show clear differences between root, stump, branch, and stem decomposition rates, probably owing to the difference in species [57, 58, 59].

### Unknown factors

Because we could not measure respiration at the same temperature and prepare samples with the same diameter ranges between positions, there were correlations between temperature and position, diameter and temperature, position and diameter, and wood density and temperature (gray lines in Fig 3). As these correlations would have been caused by our experimental design, we excluded these paths from consideration.

Despite the same decomposition period and the same sample trees, the seven factors except temperature could explain only 31% of the variance in CFWD respiration rate in the path model (Fig 3). The small *R* ^2^ value suggests that other factors were responsible for the variance. One possible factor would be microbial, such as fungal species composition, biomass, and activity. For example, logs inoculated with decay fungi had higher respiration rates than uninoculated logs [31]. Hood et al. [55] found greater fungal diversity in branches than in roots during 2- or 3-year decomposition experiments, probably due to the difference in the type of fungal colonization between airborne spores and mycelial growth in soil. A recent measurement of respiration rate using inocula to showed metabolic changes associated with antagonism between fungal species [60]. The lag period would also be a candidate, especially in the initial stage [16, 23, 43, 57]. Although the decay class of all of samples showed little visible variation, the decomposition stage, determined by weight loss or by wood density decrease, would be important to clarify the variation in respiration rate.

## Conclusion

The rate of CFWD respiration after 4 years’ decomposition on the ground and in the soil was partially accounted for by biological, physical, and chemical properties of CFWD and by environmental factors. The path model elucidated significant positive or negative relationships between variables. Although the interrelationships between explanatory variables are complex, the construction of a comprehensive model expressing these paths would allow a process-based model of CFWD decomposition to be developed. The results of this study describe the variables that influenced decomposition 4 years after felling, relatively early in the process of decomposition. Because the interrelationships would change with the progress of decomposition, similar studies will be needed at intermediate and later stages to explain the whole CFWD decomposition process.

## Supporting Information

S1 Appendix Raw data for this study, comprising soil organic matter, TN, C/N, microorganism quantity, soil moisture, soil temperature, and soil respiration rate. (XLSX)
